# Lifestyle modification program, LIFE is LIGHT, in patients with type 2 diabetes mellitus and obesity: Results from a 48‐week, multicenter, non‐randomized, parallel‐group, open‐label study

**DOI:** 10.1002/osp4.502

**Published:** 2021-04-09

**Authors:** Gagik R. Galstyan, Farida V. Valeeva, Svetlana I. Motkova, Elena V. Surkova, Larisa V. Savelyeva, Larisa M. Rudina, Krishnan Ramanathan, Ekaterina Sokareva, Kristina Bondareva, Marina V. Shestakova

**Affiliations:** ^1^ Endocrinology Research Centre Moscow Russia; ^2^ Kazan State Medical University Kazan Russia; ^3^ The Russian Presidential Academy of National Economy and Public Administration Moscow Russia; ^4^ Novartis Pharma Basel Switzerland; ^5^ Novartis Pharma LLC Moscow Russia; ^6^ Almedis Moscow Russia

**Keywords:** lifestyle modification, obesity, overweight, type 2 diabetes mellitus

## Abstract

**Background:**

Obesity is a potential risk factor for development of type 2 diabetes mellitus (T2DM). To achieve long‐term weight reduction in patients with T2DM and obesity using comprehensive lifestyle management program (LMP).

**Materials and methods:**

This 48‐week interventional, multicenter, parallel‐group, open‐label study included patients aged ≥18 years with T2DM and a body mass index (BMI) of 27–40 kg/m^2^. The primary objective was to demonstrate a clinically significant weight reduction (≥5%) from baseline in intensive lifestyle modification (ILM) and standard treatment (ST) groups.

**Results:**

The ILM group (*N* = 100) received recommendations for dietary and physical activity, and behavioral counseling. The ST group (*N* = 30) was managed in accordance with routine T2DM clinical practice. The patients in ST group were older (60.6 ± 8.9 vs. 54.6 ± 10.2 years in ILM group); overall more than 60% were women. At Week 48, the mean reduction in body weight was 5.8% (95% confidence interval [CI]: −6.9, −4.6) and 1.2% (95% CI: −2.6, 0.2) (*p* < 0.001) in the ILM and ST group, respectively. At Week 48, a weight loss of ≥5% was achieved by 50% of patients in the ILM group versus 13.3% in the ST group (*p* = 0.002). The decreases in BMI, waist‐to‐hip ratio and glycated hemoglobin (HbA1c) was significantly greater in the ILM versus ST group with between‐group differences of −1.63 (*p* ≤ 0.001), −0.03 (*р* ≤ 0.001) and −0.69% (*p* = 0.002), respectively.

**Conclusion:**

A clinically significant weight reduction (≥5%) was demonstrated in patients with obesity and T2DM with use of a comprehensive LMP, along with improvements in BMI, waist‐to‐hip ratio, and HbA1c.

## 1 INTRODUCTION

Obesity and type 2 diabetes mellitus (T2DM), the two major lifestyle disorders have a profound impact on healthcare expenditure globally due to their increasing prevalence. Obesity increases the risk of developing T2DM by 80%–85%^1^; and by 2025 around 300 million people will be affected by obesity‐related diabetes.^2^ In Russia, findings from the NATION study demonstrated increased T2DM prevalence with increasing body mass index (BMI) and obesity; in the group with a BMI less than 25 kg/m^2^ the prevalence of T2DM was 1.1%, which increased markedly to 12.0% in persons with obesity.^3^

The prevention and treatment of obesity includes several strategies such as lifestyle management (diet and physical activity), behavioral and psychological therapies, pharmaceutical interventions and bariatric surgery.^4^ Furthermore, guidelines recommend such strategies as part of diabetes self‐management education and support. Bariatric surgery has also been recommended for adults with T2DM with a BMI ≥ 40.0 kg/m^2^ (BMI ≥ 37.5 kg/m^2^ in people of Asian ancestry).^5^

Studies have demonstrated that a significantly meaningful weight loss of 5% can result in the reduction of diabetes‐related complications, thereby improving cardiovascular (CV) outcomes.^6^, ^7^, ^8^ Intensive lifestyle interventions that focus on weight management in patients with T2DM have resulted in weight loss as well as improved glycemic control and reduced risk of CV disease, as evidenced in the Action for Health in Diabetes (Look AHEAD) trial (follow‐up of 13.5 years).^6^, ^7^ Furthermore, findings from the Diabetes Remission Clinical Trial (DiRECT) study revealed that intensive weight management for 12 months within a primary care setting resulted in remission of diabetes and discontinuation of antidiabetic drugs in almost 50% of participants.^8^

Despite the knowledge on the benefits of weight loss for managing T2DM effectively, implementing and maintaining lifestyle management interventions can be a challenge for patients due to various reasons including a lack of proactive discussions between healthcare providers and patients.^9^, ^10^ This leads to unwillingness of the patients to change their habits and lifestyle, especially when they do not feel sick, or they lack the tools to assist with a long‐term change of obesity‐related parameters.^11^, ^12^ On the other hand, the use of sulphonylureas and insulin for the treatment of T2DM can result in weight gain, which is a big challenge in individuals with obesity and may result in delaying treatment intensification, leading to clinical inertia. This emphasizes the need for individualization and intensive lifestyle intervention in patients with T2DM and obesity.^13^

Over the past few decades, experience with various lifestyle modification programs in patients with T2DM and obesity across different countries such as the Diabetes Prevention Program (DPP), Look AHEAD and Practice‐based Opportunities for Weight Reduction (POWER) (USA); Malmö (Sweden); Da Qing (China); Diabetes Prevention Study (Finland and India) and a study in the Japanese population has been promising.^14^, ^15^, ^16^, ^17^, ^18^, ^19^, ^20^, ^21^ Although the findings from these programs were important, most of them were controlled studies. On the other hand, the 12‐week multidisciplinary program, Weight Achievement and Intensive Treatment (Why WAIT) was developed for use in routine clinical practice by the Joslin Diabetes Center (Boston, MA, USA) in patients with diabetes and obesity. It was a structured lifestyle intervention program, which included intensive and interactive medication adjustments, a structured modified dietary regimen, graded‐balanced and individualized exercise intervention, cognitive behavioral support and group education. The program demonstrated a marked weight loss, leading to a reduction in glycated hemoglobin (HbA1c) and blood pressure (BP) in patients with T2DM.^22^, ^23^, ^24^ However, in Russia, there is a lack of evidence supporting the benefits of such lifestyle interventional programs.

The current study LIFE is LIGHT is a 48‐week multidisciplinary program, taking inspiration from the Why WAIT. The aim of the study was to achieve long‐term weight loss in patients with T2DM and obesity using a comprehensive and holistic lifestyle change approach, thereby improving glycemic control and lipid metabolism and reducing BP levels alongside decreasing the hospitalization rate.

## 2 MATERIALS AND METHODS

### 2.1 Study design and treatment

This was a 48‐week interventional, multicenter, nonrandomized, parallel‐group, open‐label study. The patients of intensive lifestyle modification (ILM) group were assigned activities of the lifestyle management program (LMP) whereas those in standard treatment (ST) group were managed based on routine clinical practice for T2DM. All participants in the study received drug therapy for diabetes according to routine clinical practice and in accordance with the approved instructions for medical use and only for the registered indications. The treatment could be modified based on medical indications and the judgment of a physician in accordance with Russian guidelines. The study complied with the guidelines for medical care provision for patients with T2DM in Russia.

The study included a unique program of weight management, specifically designed for patients with T2DM, with five components: planned diet modification, balanced and personalized physical exercises, short‐term behavioral counseling, medical assistance, and group education. The program of active weight loss included group sessions with a team consisting of a nutritional specialist, physical therapist, clinical psychologist, and endocrinologist.

#### 2.1.1 Planned diet modification

Patients with T2DM (overweight/obese and not receiving insulin) were on a moderately hypocaloric diet with a caloric deficit of 500–1000 calories per day, but not less than 1500 kcal/day (men) and 1200 kcal/day (women). Patients were recommended to exclude or limit the consumption of animal fats and carbohydrates with high a glycemic index as much as possible, consume proteins and starches in an amount that was half of their usual intake and include foods rich in mono‐ and polyunsaturated fatty acids in their diet. Healthy carbohydrates included vegetables, wholegrain products, low‐fat dairy products and fruits. Food substitutes were not used in the study.

#### 2.1.2 Balanced and personalized physical exercises

Patients were also recommended to increase their aerobic physical activity (walking, сycling, swimming, and skiing) to 40–60 min/day. Exercise sessions under the supervision of an exercise physiologist were held once per week: this was for 30 min in the first month of the program and for 60 min in the second and third months. From the fourth month of the program, patients trained independently by taking into account the knowledge they received and the skills they developed. The exercise plan included a balanced combination of aerobic (to stimulate the development and maintain CV health), resistance (to increase muscle strength and improve productivity in everyday life) and flexibility (to improve functionality and reduce the risk of injury) exercises.

#### 2.1.3 Short‐term behavioral counseling

The components of the behavioral counseling were goal setting, self‐monitoring, control of stimulus, attributive style modification, stress management, and relapse prevention.

#### 2.1.4 Medical assistance

The endocrinologist assessed the adherence of patients to this diet plan and physical activity by using a self‐monitoring diary that the patients filled out daily for 48 weeks.

#### 2.1.5 Group education

Education classes were conducted weekly in the first 12 weeks and were followed up with monitoring once every 4 weeks for the next 36 weeks, with a total observation period of 48 weeks. Education was compliant with the principles provided in the “Standards of Specialized Diabetes Care in the Russian Federation.”^25^

During the first 12 weeks, the status of patients in the ILM group was monitored weekly (the allowed window for visits was ±1 day). After the first 13 weeks, body weight and metabolic parameters were evaluated on monthly basis. The patients in the ST group were examined for all criteria at the beginning of the study by physicians at Weeks 12, 24, 36, and 48 in order to collect data on primary and secondary endpoints.

### 2.2 Study sample

Men and women aged ≥18 years with a confirmed diagnosis of T2DM with a BMI between 27 and 40 kg/m^2^ were enrolled in the study. Written informed consent from the participant was required before any assessment was performed, along with signed informed consent from an ophthalmologist and cardiologist regarding the inclusion of the patient in the study. Pregnant or nursing (lactating) women; patients with type 1 diabetes, proliferative retinopathy, hemorrhage and disinsertion of retina, or renal impairment (serum creatinine > 1.5 mg/dL, creatinine clearance < 40 ml/min and/or proteinuria) and people with chronic alcoholism or acute alcoholic intoxication were not included into the study. Alcoholism was assessed by the investigator inquiring patients about daily amount of alcohol intake, as well as by using the medical history of the patient. Inability to perform the physical exercises due to orthopedic or CV disorders was a specific exclusion criterion for the ILM group.

### 2.3 Study objectives

The primary objective of the study was to demonstrate that an intensive lifestyle change program for patients with T2DM and obesity could lead to clinically significant weight reduction (≥5%); this was done by estimating the proportion of patients achieving this weight loss compared with baseline over a period of 48 weeks in the ILM and ST groups.

The secondary objective was to demonstrate that an intensive lifestyle change program for patients with T2DM could lead to: (a) weight reduction compared with baseline in 12, 24, 36 weeks of observation; (b) improved glycemic control as indicated by HbA1c and fasting blood glucose (FBG) levels; (c) improved BP levels; (d) improved lipid profile; and (e) improved quality of life (QoL).

Other secondary objectives included the investigation of anthropometric parameters (waist circumference, waist‐to‐hip ratio, and BMI) in both the ILM and ST groups.

### 2.4 Study assessments

The metabolic and functional status of the participants was evaluated by estimation of parameters such as BMI and body weight, waist circumference, waist‐to‐hip ratio, HBA1c, FBG, BP (systolic BP [SBP] and diastolic BP [DBP]), lipid profile (cholesterol, triglycerides, low‐density lipoprotein [LDL], and high‐density lipoprotein) and the T2DM‐related hospitalizations. The evaluation of QoL was based on the Novartis Hypoglycemia Perspectives Questionnaire and the assessment of the perceived exertion rate with the Borg Scale.^26^ The questionnaire included seven aspects of QoL: concern of symptoms related to blood glucose reduction, emotional response to the event, behavior to prevent hypoglycemia, assessment of likelihood of hypoglycemia in the future, anxiety regarding hypoglycemia, anxiety related to control of hypoglycemia, and self‐diagnosis of hypoglycemia for symptoms.

### 2.5 Sample size

Sample size was calculated based on observed weight changes in the “Why WAIT” program. The average weight loss observed at the end of 1 year was 8%. Given that the baseline criteria and translation of the program could be different when executing it in Russia, an achievable change in weight of 5% was assumed. A sample size (with a possible dropout of 20%) of 100 patients in the ILM group and 30 patients in the ST group was required to provide 80% power to detect a significant difference (significance level, *α* = 0.05) between values in the two groups.

### 2.6 Statistical analysis

The patients' disposition, demographic data, study parameters and their changes were summarized using descriptive statistics. The primary efficacy analysis was conducted in the full analysis set (FAS). The last observation carried forward method was used in the FAS in order to impute missing values of the efficacy parameters. The AE data were presented as absolute numbers and as the proportion of the patients with any AE by system organ class and preferred term according to the Medical Dictionary for Regulatory Activities. The analysis of the primary endpoint—decrease in body weight of ≥5% at 48 weeks was performed using multivariate logistic regression by calculating the odds ratio (OR) with the corresponding 95% confidence interval (CI). Confounding factors such as age, gender, baseline BMI, disease duration, and the baseline HbA1c were taken into account in the regression model. The efficacy parameters at all planned time points were compared between groups using the generalized estimating equations method taking into account the correlation between the repeated measurements.

### 2.7 Ethical considerations

The study was conducted in accordance with Good Clinical Practice and the ethical principles of the Declaration of Helsinki. An independent Ethics Committee or Institutional Review Board approved all study protocols and amendments. Informed consent forms were designed in the Russian language and were signed before including patients in the study.

## 3 RESULTS

### 3.1 Enrollment and retention

**FIGURE 1:**
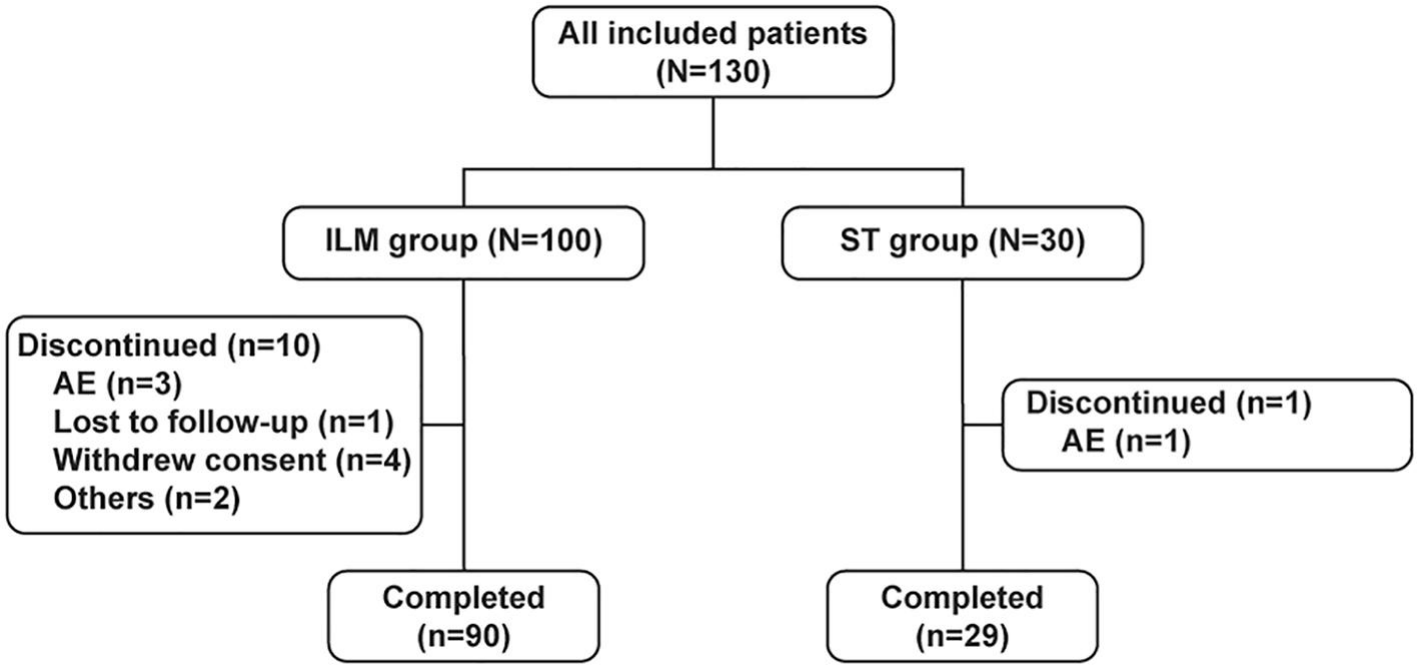
Patient disposition. AE, adverse event; ILM, intensive lifestyle modification; ST, standard treatment

Of the 130 patients enrolled in the study, 100 were included in the ILM group while the remaining 30 were included in the ST group. In the ILM group, 90 (90%) patients completed the study while 29 (96.7%) in the ST group completed the study in accordance with the protocol (Figure 1).

### 3.2 Baseline demographics and patient characteristics

Patient demographics and baseline characteristics are presented in Table 1. The proportion of women across the ILM (69.0%) and the ST (63.3%) groups were higher than the proportions of men. The mean ± *SD* age of patients in the ILM group was 54.6 ± 10.2 years whereas patients in the ST group were slightly older with a mean ± *SD* age of 60.6 ± 8.9 years. The majority of patients were Caucasian. The median duration of T2DM was 5.1 years in the ILM group and 9.3 years in the ST group. The mean ± *SD* BMI was comparable in both groups: 34.4 ± 3.53 kg/m^2^ in the ILM group and 33.5 ± 3.51 kg/m^2^ in the ST group. The mean HbA1c levels were 7.4 ± 1.74% (ILM group) and 7.8 ± 1.92% (ST group).

**TABLE 1:** Baseline demographics and patient characteristics

Demographics/Characteristics	ILM group (*N* = 100)	ST group (*N* = 30)
Age (years), mean ± *SD*	54.6 ± 10.2	60.6 ± 8.9
Gender		
Men	31.0 (31.0)	11.0 (36.7)
Women	69.0 (69.0)	19.0 (63.3)
Race		
Caucasian	99.0 (99.0)	29.0 (96.7)
Asian	1.0 (1.0)	1.0 (3.3)
Disease duration (years), mean ± *SD*	6.59 ± 5.61	11.23 ± 8.52
Weight (kg), mean ± *SD*	95.1 ± 13.3	93.3 ± 15.1
BMI (kg/m^2^), mean ± *SD*	34.39 ± 3.53	33.53 ± 3.51
HbA1c (%), mean ± *SD*	7.44 ± 1.74	7.79 ± 1.93
FBG (mmol/L), mean ± *SD*	7.41 ± 2.19	9.36 ± 3.78
SBP/DBP (mmHg), mean ± *SD*	134.8 ± 14.4/86.4 ± 9.8	131.7 ± 14.0/81.7 ± 8.9
Diabetic complications		
Microangiopathy	21.0 (21.0)	12.0 (40.0)
Macroangiopathy	18.0 (18.0)	5.0 (16.7)
Retinopathy	22.0 (22.0)	8.0 (26.7)
Nephropathy	4.0 (4.0)	3.0 (10.0)
Polyneuropathy	38.0 (38.0)	20 (66.7)
Diabetic foot	0 (0)	1 (3.3)
Other	2.0 (2.0)	2 (6.7)

*Note*: Data are presented as *n* (%) unless otherwise specified.

Abbreviations: BMI, body mass index; DBP, diastolic blood pressure; FBG, fasting blood glucose; HbA1c, glycated hemoglobin; ILM, intensive lifestyle modification; SBP, systolic blood pressure; *SD*, standard deviation; ST, standard treatment.

### 3.3 Primary efficacy parameters

**FIGURE 2:**
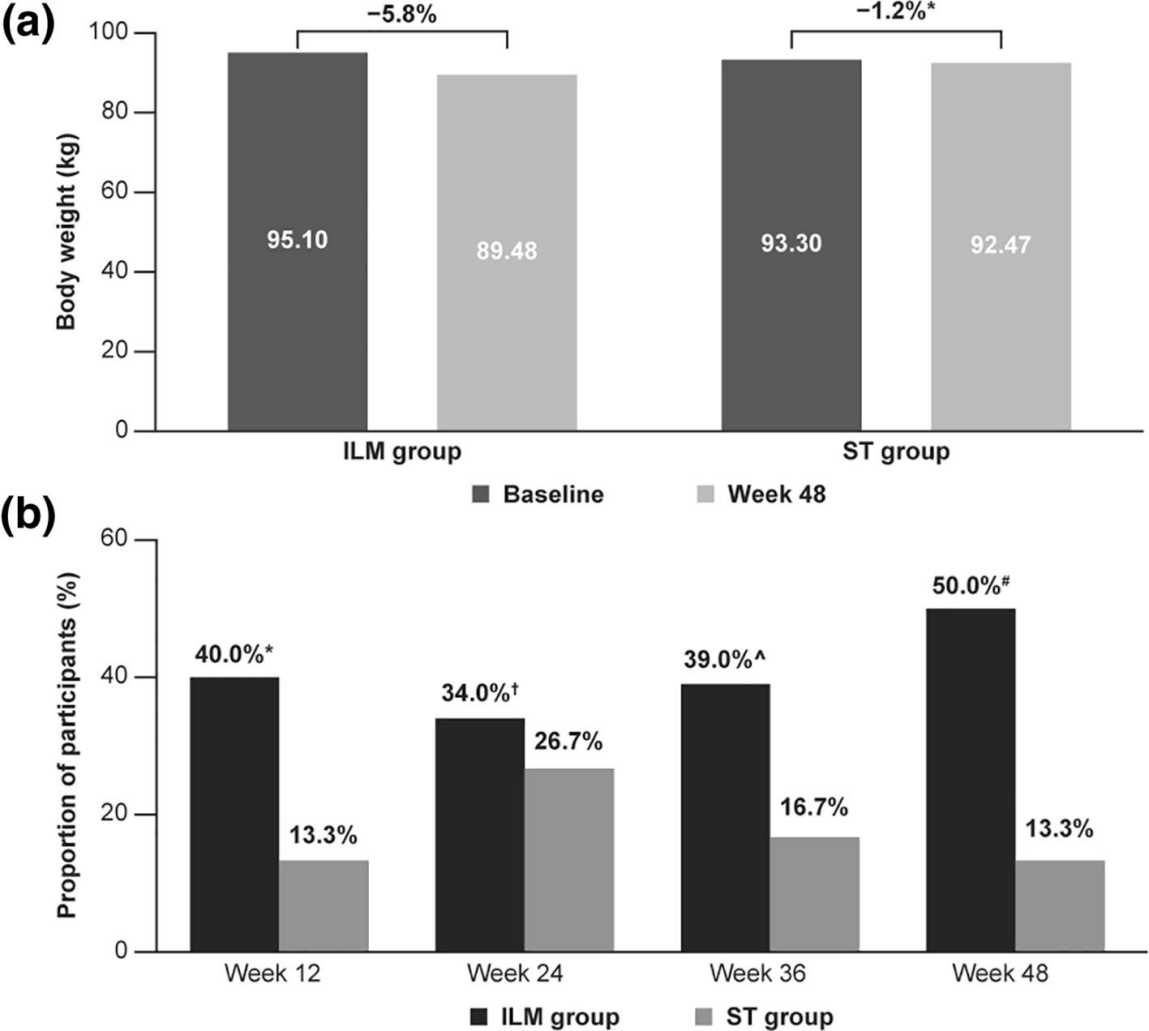
Change in body weight from baseline to Week 48 (A) and proportion of patients achieving a 5% decrease in body weight by study visit (B)

**FIGURE 3:**
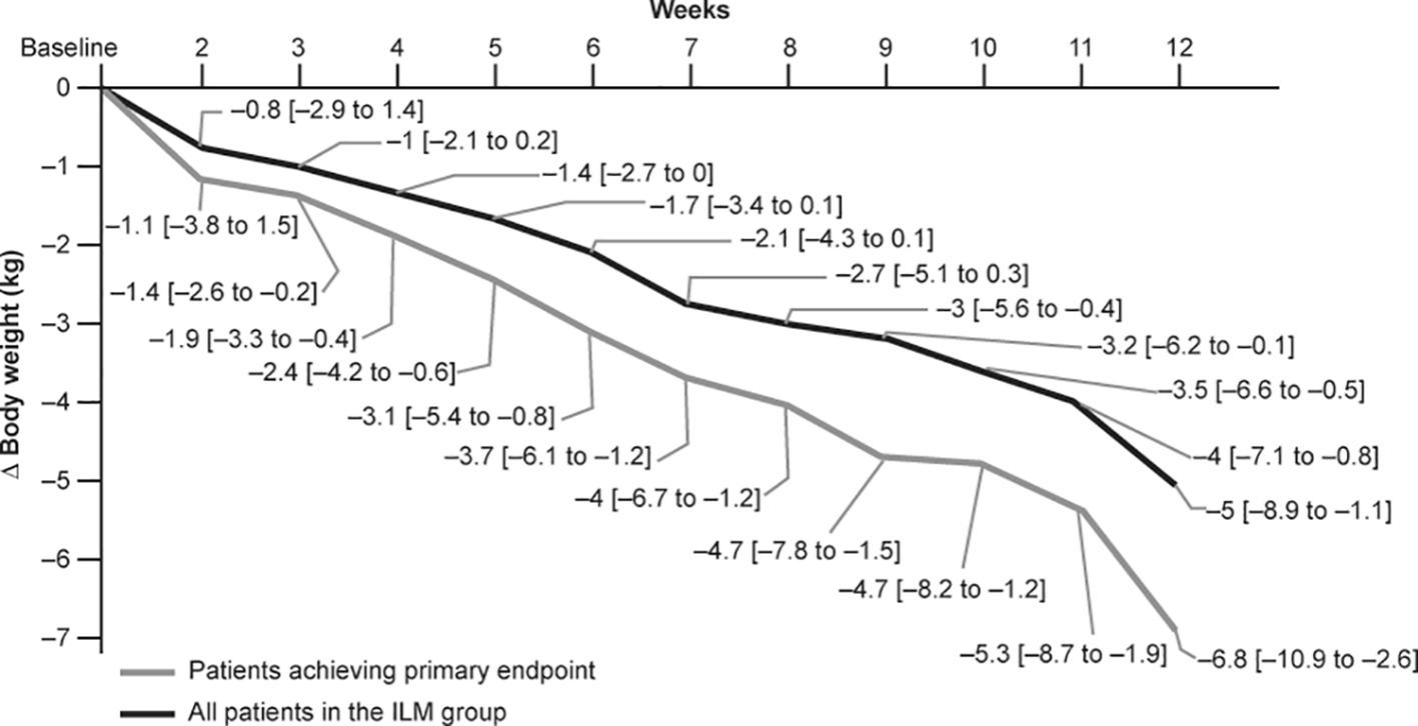
Reduction in body weight in patients of the ILM group, including those who achieved the primary endpoint (weight loss ≥ 5%), in the first 3 months of program (mean [95% CI]). CI, confidence interval; ILM, intensive lifestyle management. Adapted from Motkova et al.^27^

Compared with baseline, patients in the ILM group lost 5.8% (95% CI: −6.9, −4.6) while those in the ST group lost 1.2% (95% CI: −2.6, 0.2) of body weight at Week 48 (Figure 2A). A weight reduction of ≥5% by the end of the study was achieved by 50% (95% CI: 40.3, 59.7) of patients in the ILM group and by 13.3% (95% CI: 5.1, 30.6) in the ST group (Figure 2B). The odds of a decrease in body weight of ≥5% at Week 48 of the follow‐up were statistically significantly higher among the patients in the ILM group compared with those in the ST group (OR: 6.54; 95% CI: 2.01, 21.33; *p* = 0.002). The reduction in body weight in patients of the ILM group, including those who achieved the primary endpoint (weight loss ≥5%), in the first 3 months of program has been presented in Figure 3.^27^

### 3.4 Secondary efficacy parameters

#### 3.4.1 Body weight

**FIGURE 4:**
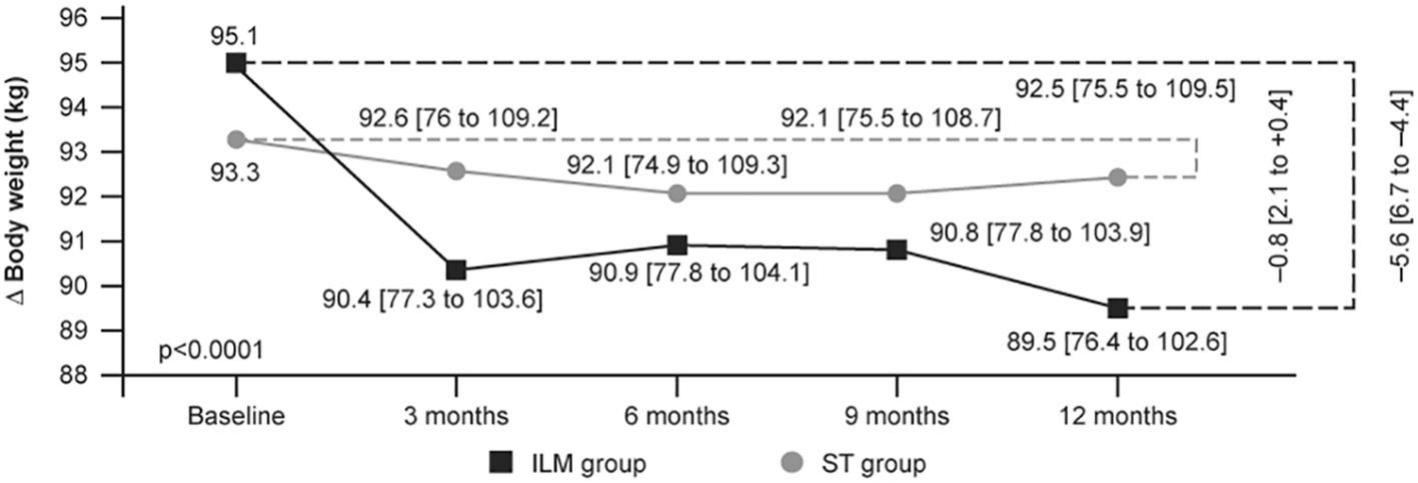
Reduction in body weight during the study (mean [95% CI]). CI, confidence interval; ILM, intensive lifestyle management; ST, standard treatment. Adapted from Motkova et al.^27^

A mean ± *SE* weight reduction of 4.84 ± 0.38% was observed in the ILM group at Week 12 from the start of the study. However, the mean ± *SE* weight loss at Week 24 (4.24 ± 0.53%) and 36 (4.31 ± 0.56%) in the ILM group remained unchanged compared to baseline. There was a slight or no decrease in body weight in the ST group at Week 12 (0.91 ± 0.69%), Week 24 (1.57 ± 0.79%), and Week 36 (1.48 ± 0.72%) compared to baseline. Overall, a steady and significant decrease in body weight was observed in the ILM versus control group from baseline until end of the study (Figure 4).^27^ The percentage changes in body weight from baseline to Week 12, 24, 36, and 48 and the corresponding between group comparisons are presented in Table 2.

**TABLE 2:** Changes in the secondary efficacy parameters from baseline to Weeks 12, 24, 36, and 48 of the study period

Parameters	Week 12	Week 24	Week 36	Week 48
Body weight (%)				
ILM group (*N* = 100)	−4.84 ± 0.38	−4.24 ± 0.53	−4.31 ± 0.56	−5.75 ± 0.59
ST group (*N* = 30)	−0.91 ± 0.69	−1.57 ± 0.79	−1.48 ± 0.72	−1.15 ± 0.71
Between‐group difference	−3.93 ± 0.79	−2.66 ± 0.95	−2.82 ± 0.91	−4.59 ± 0.92
95% CI (LL, UL); *p* value	−5.47, −2.38; *p* < 0.001	−4.53, −0.80; *p* = 0.005	−4.61, −1.04; *p* = 0.002	−6.40, −2.78; *p* < 0.001
BMI (kg/m^2^)				
ILM group (*N* = 100)	−1.66 ± 0.13	−1.46 ± 0.18	−1.48 ± 0.19	−1.97 ± 0.20
ST group (*n* = 30)	−0.28 ± 0.22	−0.49 ± 0.26	−0.47 ± 0.23	−0.34 ± 0.23
Between‐group difference	−1.38 ± 0.26	−0.98 ± 0.31	−1.02 ± 0.30	−1.63 ± 0.30
95% CI (LL, UL); *p* value	−1.89, −0.87; *p* < 0.001	−1.59, −0.36; *p* = 0.002	−1.61, −0.43; *p* < 0.001	−2.22, −1.04; *p* < 0.001
Waist‐to‐hip ratio				
ILM group (*N* = 100)	−0.02 ± 0.003	−0.03 ± 0.004	−0.02 ± 0.004	−0.02 ± 0.004
ST group (*N* = 30)	0.006 ± 0.004	0.01 ± 0.005	0.01 ± 0.005	0.02 ± 0.005
Between‐group difference	−0.02 ± 0.01	−0.04 ± 0.006	−0.03 ± 0.007	−0.03 ± 0.006
95% CI (LL, UL); *p* value	−0.03, −0.01; *p* < 0.001	−0.05, −0.02; *p* < 0.001	−0.05, −0.02; *p* < 0.001	−0.04, −0.02; *p* < 0.001
Waist circumference (cm)				
ILM group (*N* = 100)	−6.2 ± 0.47	−6.1 ± 0.52	−5.8 ± 0.53	−7.0 ± 0.59
ST group (*N* = 30)	0.0 ± 0.53	−0.1 ± 0.63	0.2 ± 0.58	0.2 ± 0.60
Between group difference	−6.2 ± 0.71	−6.0 ± 0.81	−6.1 ± 0.78	−7.2 ± 0.84
95% CI (LL, UL); *p* value	−7.6, −4.8; *p* < 0.001	−7.6, −4.4; *p* < 0.001	−7.6, −4.5; *p* < 0.001	−8.9, −5.6; *p* < 0.001
HbA1c (%)				
ILM group (*N* = 100)	−0.69 ± 0.14	−0.75 ± 0.15	−0.71 ± 0.14	−0.79 ± 0.14
ST group (*N* = 30)	−0.25 ± 0.16	−0.45 ± 0.17	−0.07 ± 0.20	−0.11 ± 0.17
Between‐group difference	−0.44 ± 0.21	−0.30 ± 0.23	−0.64 ± 0.24	−0.69 ± 0.22
95% CI (LL, UL); *p* value	−0.86, −0.03; *p* = 0.036	−0.74, −0.15; *p* = 0.189	−1.12, −0.16; *p* = 0.009	−1.12, −0.25; *p* = 0.002

*Note*: Data are represented as mean ± *SE* unless otherwise specified.

Abbreviations: BMI, body mass index; CI, confidence interval; HbA1c, glycated hemoglobin; ILM, intensive lifestyle modification; LL, lower limit; *SE*, standard error; ST, standard treatment; UL, upper limit.

In the ILM group, a weight loss of ≥10% from baseline was observed in 14.0% of patients at 24 weeks, 15.0% at 36 weeks and 26.0% at 48 weeks. Among the ST group, weight loss of 10% or more was seen only in two participants (6.7%) after 24 weeks and in one participant (3.3%) at Week 48.

#### 3.4.2 BMI, waist circumference, and waist‐to‐hip ratio

The reductions in BMI and waist‐to‐hip ratio, were statistically significant in the ILM group compared with the ST group after 48 weeks of follow up. The difference in the mean ± *SE* changes of the values between groups was −1.63 ± 0.3 kg/m^2^ for BMI (*р* < 0.001) and −0.03 ± 0.01 for the waist‐to‐hip ratio (*р* < 0.001) (Table 2). Similarly, the difference in waist circumference at Week 12, 24, 36, and 48 from the start of the study was statistically significant (Table 2).

#### 3.4.3 HbA1c and FBG

**FIGURE 5:**
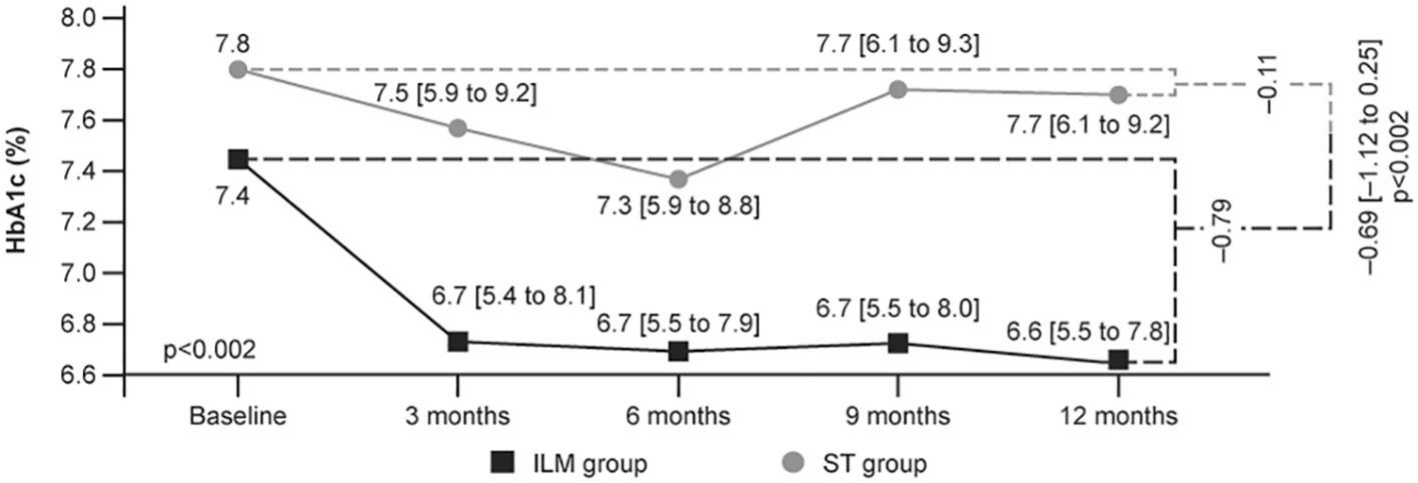
Change in HbA1c levels during the study (mean [95% CI]). CI, confidence interval; HbA1c, glycated hemoglobin; ILM, intensive lifestyle management; ST, standard treatment. Adapted from Motkova et al.^27^

The mean change in HbA1c from baseline at the end of 48 weeks was significantly greater in the ILM group than in the ST group. The difference in the mean ± *SE* changes in HbA1c values between the groups was −0.69 ± 0.22% (95% CI: −1.12, −0.25; *p* = 0.002) (Table 2). The reduction in the levels of HbA1c is depicted in Figure 5.^27^ No statistically significant difference in the FBG level was observed between the groups during the study.

#### 3.4.4 Glucose‐lowering drugs

**FIGURE 6:**
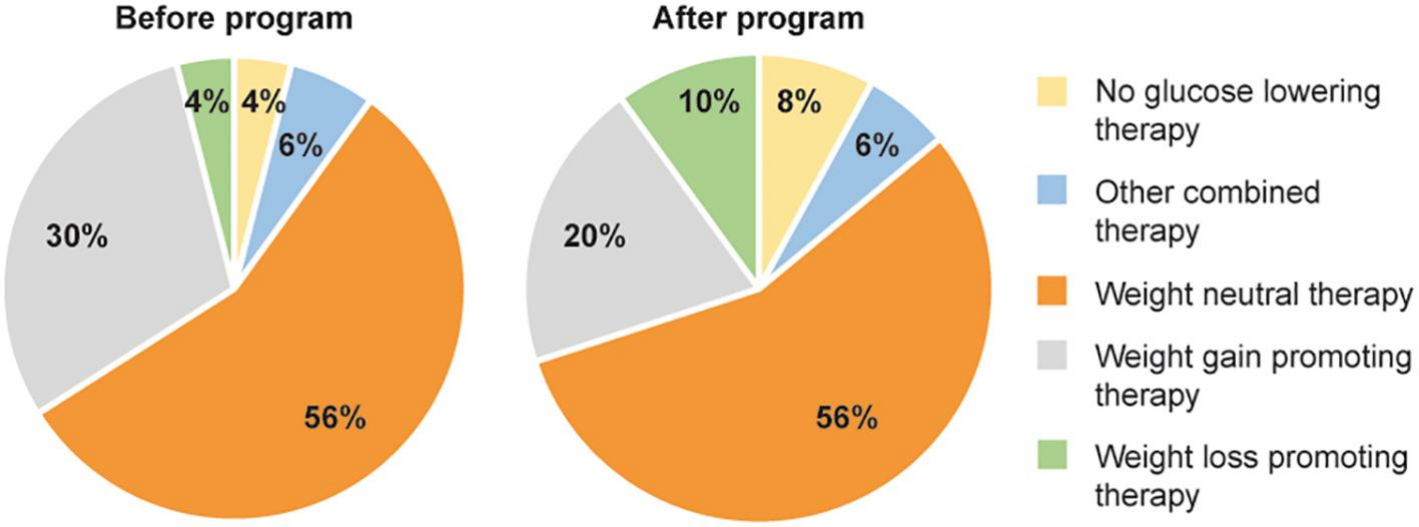
Distribution of therapies based on the weight impact of glucose‐lowering drugs used in the intensive group patients (ILM) who met the primary endpoint (before and after program interventions) Other combined therapy—Met + SU + GLP‐1 RA, GLP‐1 RA + insulin, Met + GLP‐1 RA + insulin; weight neutral therapy—Met, Met + DPP‐4i; weight gain promoting therapy—Met + (SU and/or insulin), SU + DPP‐4i, Met + (SU or insulin) + DPP‐4i; weight loss promoting therapy—Met + SGLT‐2i, Met + GLP‐1 RA. DPP‐4i, dipeptidyl peptidase‐4 inhibitors; GLP‐1 RA, glucagon‐like peptide‐1 receptor agonists; Met, metformin; SGLT‐2i, sodium‐glucose co‐transporter‐2 inhibitors; SU, sulphonylureas. Adapted from Motkova et al.^27^

Even though “LIFE is LIGHT” is an interventional program, the use of drug therapy was in accordance with routine clinical practice and for the indications listed in drug labels. All the drug prescriptions were at the discretion of the physician and not subjected to the protocol related interventions of the program. However, it is worth mentioning that comprehensive lifestyle modifications, behavioral counseling, and educational support could contribute to the structure of medications used among patients in the intensive (ILM) group before and after the program (Figure 6). Thus, patients who met the primary endpoint of the study were less likely to use drugs associated with weight gain (decrease by one‐third in use). The difference was due to both an increase (from 4% to 10%) in the use of weight loss promoting therapy and the reduced need for antidiabetes drugs (doubling from 4% to 8% of subjects with no glucose‐lowering therapy). In addition, the structure of oral antidiabetes drugs was similar in the ILM and ST groups because drugs were prescribed according to current treatment guidelines.

#### 3.4.5 BP and lipid profile

The SBP decreased, by a mean ± *SE* by 7.4 ± 1.56 mmHg (95% CI: −10.5, −4.4) in the ILM group and by 1.0 ± 2.64 mmHg (95% CI: −6.1, −4.4) in the ST group at the end of the 48‐week follow‐up period. The difference in the mean changes in SBP values between the groups was significant (mean ± *SE*: −6.5 ± 3.07; 95% CI: −12.5, −0.4; *p* = 0.035). Although the DBP was lower in the ILM compared with the ST group at 36 weeks, there was no statistically significant difference in mean DBP changes between the groups (mean ± *SE*: −2.0 ± 1.52; 95% CI: −5.0, 1.0; *p* = 0.184) at the end of 48 weeks. Changes in the lipid profile did not differ significantly between the ILM and ST groups during the study.

#### 3.4.6 Self‐report of hypoglycemia (questionnaire based)

Patients completed a questionnaire to assess hypoglycemia at baseline, Week 24 and 48 of follow‐up. At baseline, 70.0% (*n*/*N*, 70/100) of patients in the ILM group and 73.3% (*n*/*N*, 22/30) in the ST group reported that they had never experienced episodes of hypoglycemia. After 24 weeks, the proportion of such patients in the ILM and ST groups was 59.6% (*n*/*N*, 53/89) and 60.0% (*n*/*N*, 18/30), respectively. At the end of the study (48 weeks), 54.4% (*n*/*N*, 49/90) of patients in the ILM group and 62.1% (*n*/*N*, 18/29) of patients in the ST group who completed the study reported no hypoglycemic episodes.

Patients in the ST group did not report hypoglycemic events within a year prior to the study or during the study. In the ILM group, one patient was hospitalized due to a decrease in the blood glucose level; one patient required emergency medical help twice; three patients needed help from another person several times; and one patient lost consciousness twice.

#### 3.4.7 QoL and physical activity assessments

The mean values of the scores for the seven aspects of the QoL questionnaire were comparable in both groups. The score did not exceed 5 in the ILM group, while in the ST group, the average score exceeded 5 points only in 2 aspects: likelihood of hypoglycemia in the future and symptomology.

The mean values of the assessment of physical activity by patients in the ILM group on the Borg Scale were similar after the initial training (3.3) and at the end (3.4) of the program.

## 4 DISCUSSION

The LIFE is LIGHT study demonstrated that after 48 weeks of a comprehensive lifestyle change program, a clinically significant weight loss of ≥5% was observed in 50% of the participants in the ILM group compared to 13.3% in the ST group. The program comprised of recommendation on dietary modifications and physical activity along with behavioral counseling by a team of multidisciplinary specialists. The lifestyle modifications also reduced the levels of important prognostic markers related to T2DM such as HbA1c, BMI and the waist‐to‐hip ratio.

A substantial weight loss was observed in the first 12 weeks, which reached a plateau at Weeks 24 and 36 in the ILM group. This demonstrated the sustainability of the weight loss in the ILM group at Weeks 24 and 36, which is further maintained at Week 48. However, this trend was not observed in the ST group. The findings from this study are in line with earlier studies based on intensive lifestyle interventions such as Look AHEAD,^15^, ^28^, ^29^ DiRECT,^8^ DPP,^14^ POWER,^16^ and Why WAIT^22^, ^23^, ^24^ that have also demonstrated beneficial effects of weight loss in patients with T2DM and obesity.

The results of the Why WAIT program demonstrated a marked weight loss of 5%–8%, reduced body fat and abdominal obesity, reduced BP, which led to a concomitant drop in HbA1c of 0.6%–1.0% from baseline.^23^ Similarly, the weight loss of ≥5% observed over period of 1 year in the current study are also crucial in light of the finding from the follow‐up of the Why WAIT. That follow‐up study demonstrated that patients who sustained ≥7% weight loss at 1 year were more likely to maintain significant weight loss after 5 years of follow‐up.^24^

The results from Look AHEAD, a large randomized study demonstrated greater weight loss in the intervention group (decreased caloric intake and increased physical activity) versus the control group (received diabetes support and education) throughout the study (8.6% vs. 0.7% at 1 year; 6.0% vs. 3.5% at the end of the study). This led to reduction in HbA1c and initial improvements in fitness and CV risk factors (SBP, DBP, and triglycerides) except for LDL cholesterol levels, which is in line with the current findings. Although the Look AHEAD trial did not show that an intensive lifestyle intervention reduced CV events in adults with T2DM and obesity, it did show the feasibility of achieving and maintaining long‐term weight loss in patients with T2DM.^27^ However, the post hoc analysis of the Look AHEAD showed that 85% of patients with moderately or well‐controlled HbA1c and good overall health had reduced CV events, whereas 15% of participants with well‐controlled HbA1c and poor health experienced negative effects; this may have resulted in an overall neutral outcome of the study.^28^

In addition, the DPP study highlights that the intensive lifestyle intervention led to marked weight loss (5.6 vs. 2.1 kg) and reduction in incidence of diabetes (7.8 cases vs. 4.8 cases per 100 person‐years) when compared to standard‐of‐treatment, metformin.^14^ Another study (POWER) which included patients with obesity with at least one CV risk factor demonstrated a sustained clinically significant weight loss over a period of 24 months irrespective of the models of lifestyle management (in‐person vs. remote support). The percentage of participants whose weight was at least 5% below their baseline weight was 18.8% in the control group, 41.4% in the group receiving in‐person support, and 38.2% in the group receiving remote support only at the end of the study.^16^ Similarly, in the current study, the ILM and standard therapy approaches have also demonstrated a ≥5% body weight loss in 50% (95% CI: 40.3, 59.7) and 13.3% (95% CI: 5.1, 30.6) of patients, respectively, by the end of the study.

The intervention group in the LIFE is LIGHT study showed significant reduction in HbA1c levels at 48 weeks. The glycemic durability observed with ILM in this study, is in line with the DIRECT study that showed that lifestyle intervention focusing on weight management can revert the state of diabetes to nondiabetes (HbA1c < 6.5%) and can lead to discontinuation of antihyperglycemic drugs.^8^ The improvements in glycemia induced by weight loss are most likely to be seen early in the natural progression of T2DM when insulin resistance due to obesity has caused reversible *β*‐cell dysfunction but insulin secretory capacity remains relatively preserved.^30^, ^31^

Although fewer patients experienced hypoglycemia at the end of the study period, the hypoglycemic conditions were evident during the program. Considering this, it is recommended to make substantial efforts while designing lifestyle intervention programs to prevent hypoglycemic events and choose antidiabetes drugs, which do not induce hypoglycemia.

The components of the intensive lifestyle intervention program in the current study contributed to good QoL in the study participants, as evaluated by the results of hypoglycemia questionnaire and Borg Scale. As compared to baseline, there was a decrease in the proportion of patients experiencing hypoglycemia at the end of the study period. In addition, the mean values of the assessment of physical activity among patients in the ILM group were approximately the same after the first training and at the end of the program. These findings reflect that self‐managed behaviors, relationships between patients and providers, and education programs to motivate lifestyle changes help to improve QoL in patients with T2DM.^32^

The components of ILM such as balanced diet, physical activity, medical assistance, behavioral counseling, and group education have demonstrated an overall improvement in metabolic parameters such as HbA1c, BP, sustained reduction in body weight with minimal hypoglycemic events as well as good QoL in the study participants.

Obesity is an established risk factor for several noncommunicable diseases such as CV disease, T2DM, hypertension and coronary heart disease, and even certain cancers.^33^ It is the basis for the development of insulin resistance and results in carbohydrate metabolism compensation, which is associated with CV risk factors.^13^ Thus, T2DM treatment should focus on early lifestyle intervention alongside pharmacotherapy in order to reduce the risk of diabetes‐related complications. Targeting body weight is a reliable way to contribute to optimal HbA1c levels, and is a viable and cost‐effective measure for the optimal management of T2DM in routine clinical practice.

There were a few limitations to this study. One of such limitations is the lack of randomization that may induce selection bias. Another limitation was the relatively small sample size of the study population and may lead to higher variability in the observed mean values and bias.

In conclusion, the study demonstrated a clinically significant weight loss of ≥5% in patients with T2DM and obesity along with a positive impact on BMI, waist‐to‐hip ratio and HbA1c by the use of a proposed comprehensive LMP. The LIFE is LIGHT study highlights that a pragmatic approach of dietary modifications, physical activity, and behavioral counseling results in clinically significant weight loss and improved QoL.

## CONFLICT OF INTERESTS

Gagik R. Galstyan serves on advisory panels for AbbVie, AstraZeneca, Merck Sharp & Dohme, Novo Nordisk, Pfizer, and Sanofi; and is a member of speaker's bureaus for Amgen, AstraZeneca, Berlin Chemie, Boehringer Ingelheim, Eli Lilly, LifeScan, Merck Sharp & Dohme, Novartis, Novo Nordisk, Sanofi, Servier, and Takeda. Farida V. Valeeva has received speaker's honoraria and has served on advisory boards for Sanofi‐Aventis, AstraZeneca, Ascensia, Novartis, Boehringer Ingelheim, Takeda, Roche, and Medtronic. Elena V. Surkova has received speaker's honoraria and has served on advisory boards for Novo Nordisk, Sanofi‐Aventis, Eli Lilly, Johnson & Johnson, Ascensia, and Novartis. Larisa M. Rudina participated in the educational programs (provided to physicians) managed at the expense of the following companies: Novartis, Sanofi, Takeda, Recordatti, Novo Nordisk during the period of the research. Krishnan Ramanathan is an employee and owns stock of Novartis Pharma AG. Ekaterina Sokareva was an employee of Novartis Pharma LLC during the conduct of study. Marina V. Shestakova has received honoraria from Eli Lilly, Merck Sharp & Dohme, Sanofi, Novo Nordisk, Boehringer Ingelheim, AstraZeneca and Novartis and research support from Sanofi. Larisa V. Savelyeva, Svetlana I. Motkova, and Kristina Bondareva declare no conflict of interests.

## AUTHOR CONTRIBUTIONS

Gagik R. Galstyan and Elena V. Surkova contributed in developing the research concept and design, editing, and revised the manuscript for important intellectual content. Farida V. Valeeva, Ekaterina Sokareva, and Marina V. Shestakova revised the manuscript for important intellectual content and provided editing inputs. Svetlana I. Motkova, Larisa V. Savelyeva, and Larisa M. Rudina conducted classes/sessions with patients in the study and collected the clinical data. Krishnan Ramanathan revised the manuscript for important intellectual content, conducted statistical processing of research results and their interpretation and provided editing inputs. Kristina Bondareva was involved in statistical processing of research results and their interpretation. All authors interpreted the data, critically reviewed and approved the final submitted manuscript.
